# Polymerizable inhibitor can reduce shrinkage stress of resin composite

**DOI:** 10.1007/s00784-026-06901-z

**Published:** 2026-05-11

**Authors:** Jingwei He, Sufyan Garoushi, Lippo Lassila, Pekka K. Vallittu

**Affiliations:** 1 Department of Biomaterials Science and Turku Clinical Biomaterials Center-TCBC, Institute of Dentistry, University of Turku, Turku, Finland; 2 College of Materials Science and Engineering, South China University of Technology, Guangzhou, China; 3 City of Turku Welfare Division, Oral Health Care, Turku, Finland

**Keywords:** Dental resin composites, Polymerizable inhibitor, Shrinkage stress, Physicochemical properties, Leachable inhibitors

## Abstract

**Objective:**

The purpose of this study was to prepare dental resin composites (DRCs) with low shrinkage stress and limited leachable inhibitor content by incorporating a synthesized polymerizable inhibitor PBHT (2,6-di-tert-butyl-4-(methacryloyloxy-ethyl-carbamate-methyl)phenol).

**Method:**

The PBHT was synthesized via the reaction between 2,6-di-*tert*-butyl-4-hydroxymethylphenol and 2-(methacryloyloxy)ethyl isocyanate. The structure of PBHT was confirmed by FT-IR and ^1^H-NMR spectra. The PBHT was added to the resin matrix of the DRCs at mass ratios of 1, 2, and 3 wt%. Double bond conversion (DC), volumetric shrinkage (VS), shrinkage stress (SS), flexural properties, water sorption (WS) and solubility (SL) of the prepared DRCs were investigated according to standard or referenced methods. The amount of leachable inhibitors in DRCs was determined by gas chromatography-mass spectrometry using the selected ion monitoring method. For comparative purposes, the DRCs containing the commercial inhibitor butylated hydroxytoluene (BHT) were also prepared, while a DRC without any extra inhibitor was used as the control.

**Results:**

Adding PBHT to the resin matrix significantly reduced the DC of DRCs (*p* < 0.05). The PBHT lowered VS and SS of DRCs (*p* < 0.05), in contrast to BHT, which showed no significant effect (*p* > 0.05). Neither PBHT nor BHT affected flexural properties of DRCs (*p* > 0.05). The WS of DRCs was significantly increased after adding 3 wt% of either inhibitor (*p* < 0.05). While both PBHT and BHT significantly raised SL (*p* < 0.05), the PBHT-containing DRCs showed significantly lower SL than the BHT-containing ones at the same concentration (*p* < 0.05). Compared with the BHT-containing DRCs, PBHT-containing DRCs had lower leachable inhibitors (*p* < 0.05).

**Significance:**

The PBHT-containing DRCs achieved low shrinkage stress and limited amount of leachable inhibitors. At the optimal concentration of 1 wt%, PBHT had no significant effect on the physicochemical properties other than SL.

## Introduction

Due to their versatility, dental resin composites (DRCs) have been applied into dentistry as restorative materials, pit and fissure sealants, cavity liners, endodontic sealer, root canal posts, and luting materials [1, 2]. However, during the polymerization of DRCs, volumetric shrinkage and shrinkage stress that occur on the adjacent tooth structure is a problem in restorative dentistry [3]. When the shrinkage stress is concentrated sufficiently, it may eventuate in interfacial bond failure, microleakage, deformation of the tooth cusps, post-operative sensitivity, marginal staining, and secondary caries [1, 4–8].

Over the past decades, various strategies have been developed to reduce the shrinkage stress of dental resin composites (DRCs), including the use of novel monomers and advanced polymerization techniques. These approaches generally follow two main strategies: (1) reducing volumetric shrinkage [8–12] and (2) delaying the gel point [13–22]. Evidence from the literature suggests that delaying the gel point is more effective in reducing shrinkage stress than merely reducing volumetric shrinkage [23].

During the polymerization of DRCs, before reaching gel point, the shrinkage stress might be released through molecular rearrangement of polymer chain segments, leading to low stress at the adhesive interface [24]. The gel point of DRCs can be delayed through utilizing monomers that possess unique polymerization mechanisms [15–22] and by slowing down polymerization rate [13, 14, 25]. Importantly, an ideal method should not interfere with the double bond conversion and/or final performance of DRCs.

**Fig. 1:**
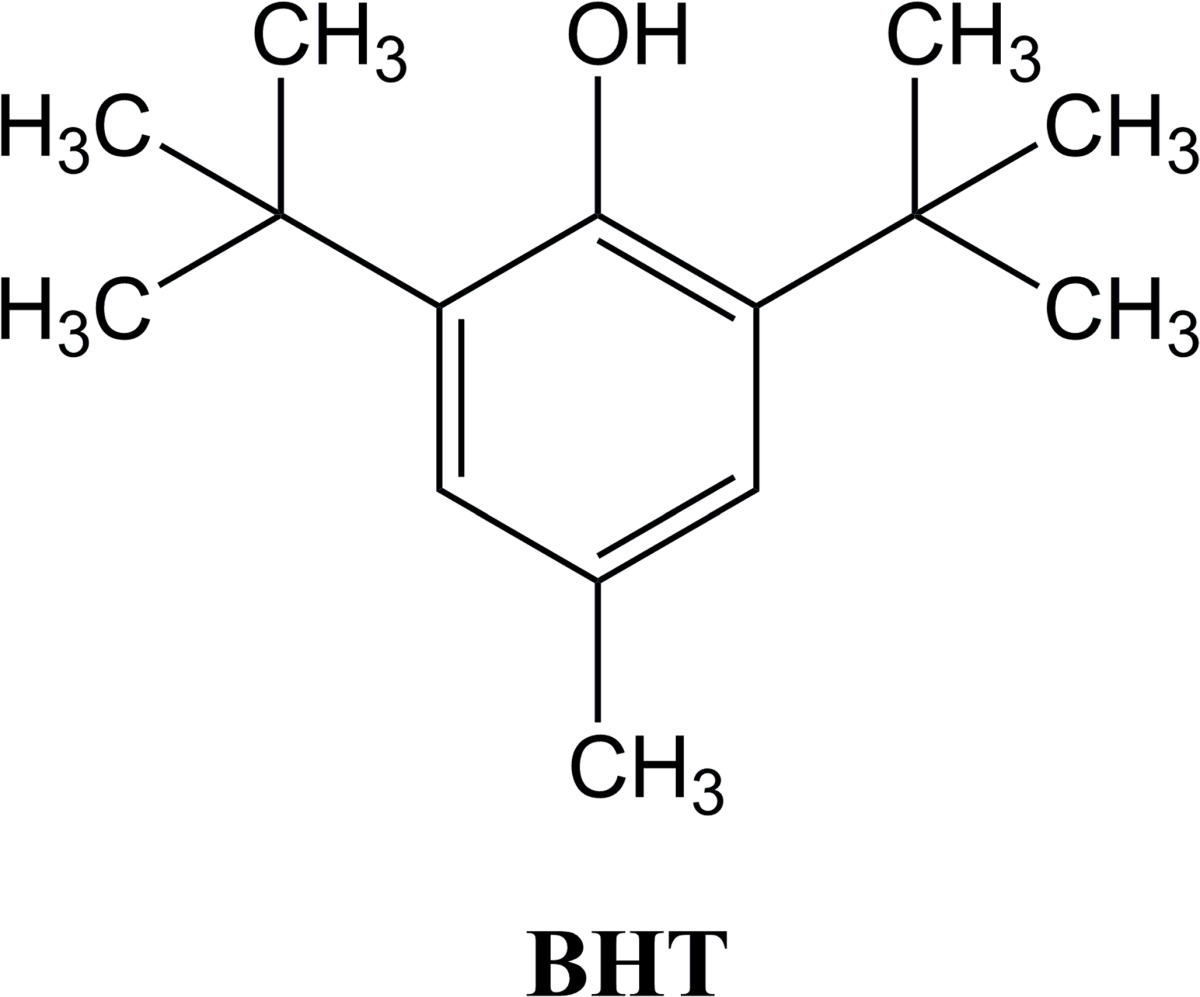
Structure of BHT

Butylated hydroxytoluene (BHT, structure is shown in Fig. 1) is a commercially synthetic antioxidant used to extend the shelf-life of food [26]. In free radical polymerization, a free radical can react with the phenolic hydrogen in BHT molecules, producing a highly stabilized, sterically hindered radical that is incapable of re-initiating chain growth. As a result, the polymerization proceeds at a reduced rate until the inhibitor is consumed. For this reason, BHT is also used in commercial DRCs to inhibit premature and spontaneous polymerization triggered by light [27–29]. The study of Braga et al. [24] showed that increasing the BHT concentration in DRCs could reduce the polymerization rate, resulting in less shrinkage stress without compromising the degree of conversion. However, Pereira et al. [30] pointed out that for the purpose of reducing shrinkage stress and maintaining physicochemical properties, the concentration of BHT in resin matrix should be less than 1 wt%.

Although BHT is widely used as a direct or indirect food/feed additive, an overdose of BHT has been reported to adversely affect various tissues and organs in rats [31–33]. As a molecule that cannot undergo polymerization reaction, BHT can be easily released out of the material. There were several elution studies showed that BHT was eluted from the polymerized DRCs [34–37]. Although the amount of BHT eluted was very low [35], its high cytotoxic potential observed in permanent 3T3 cells and three types of primary human fibroblasts derived from oral tissues (gingiva, pulp, and periodontal ligament) [38] has led to suggestion that a potentially safer compound should be used to replace BHT if possible. Thus, developing a polymerizable alternative would be even better.

In this research, a polymerizable inhibitor named 2-((((3,5-di-*tert*-butyl-4-hydroxybenzyl)oxy)carbonyl)amino)ethyl methacrylate (PBHT) was synthesized and added into DRCs for reducing shrinkage stress. The effects of PBHT on the double bond conversion, volumetric shrinkage, shrinkage stress, flexural properties, water sorption and solubility of DRCs and leachability of PBHT from DRCs were investigated. The DRCs containing commercial inhibitor BHT were used as control. The hypotheses of this study were: (1) the polymerizable inhibitor would reduce the shrinkage stress of DRCs without impairing their physicochemical properties; and (2) the amount of leachable inhibitor would be lower in DRCs containing the polymerizable inhibitor than in those containing BHT.

## Materials and methods

### Materials

2,6-di-*tert*-butyl-4-hydroxymethylphenol (HBHT), 2-(methacryloyloxy)ethyl isocyanate (MEI), dibutyltin dilaurate (DBTL), and camphorquinone (CQ) were purchased from Tokyo Chemical Industry Co., Ltd. (Tokyo, Japan). Urethane dimethacrylate (UDMA) and Bisphenol A ethoxylate (04) dimethacrylate (Bis-EMA (04)) were purchased from Esstech Inc. (Essington, PA, USA). Triethyleneglycol dimethacrylate (TEGDMA), Ethyl-4-dimethylaminobenzoate (EDB), and BHT were purchased from Sigma-Aldrich Co., Ltd. (St Louis, MO, USA). Silanated dental glass fillers (SCHOTT^®^ UltraFine, G018-053, 6% silane, 0.7 μm) were purchased from SCHOTT AG (Mainz, Germany).

### Synthesis of 2-((((3,5-di-tert-butyl-4-hydroxybenzyl)oxy)carbonyl)amino)ethyl methacrylate (PBHT)

**Fig. 2:**
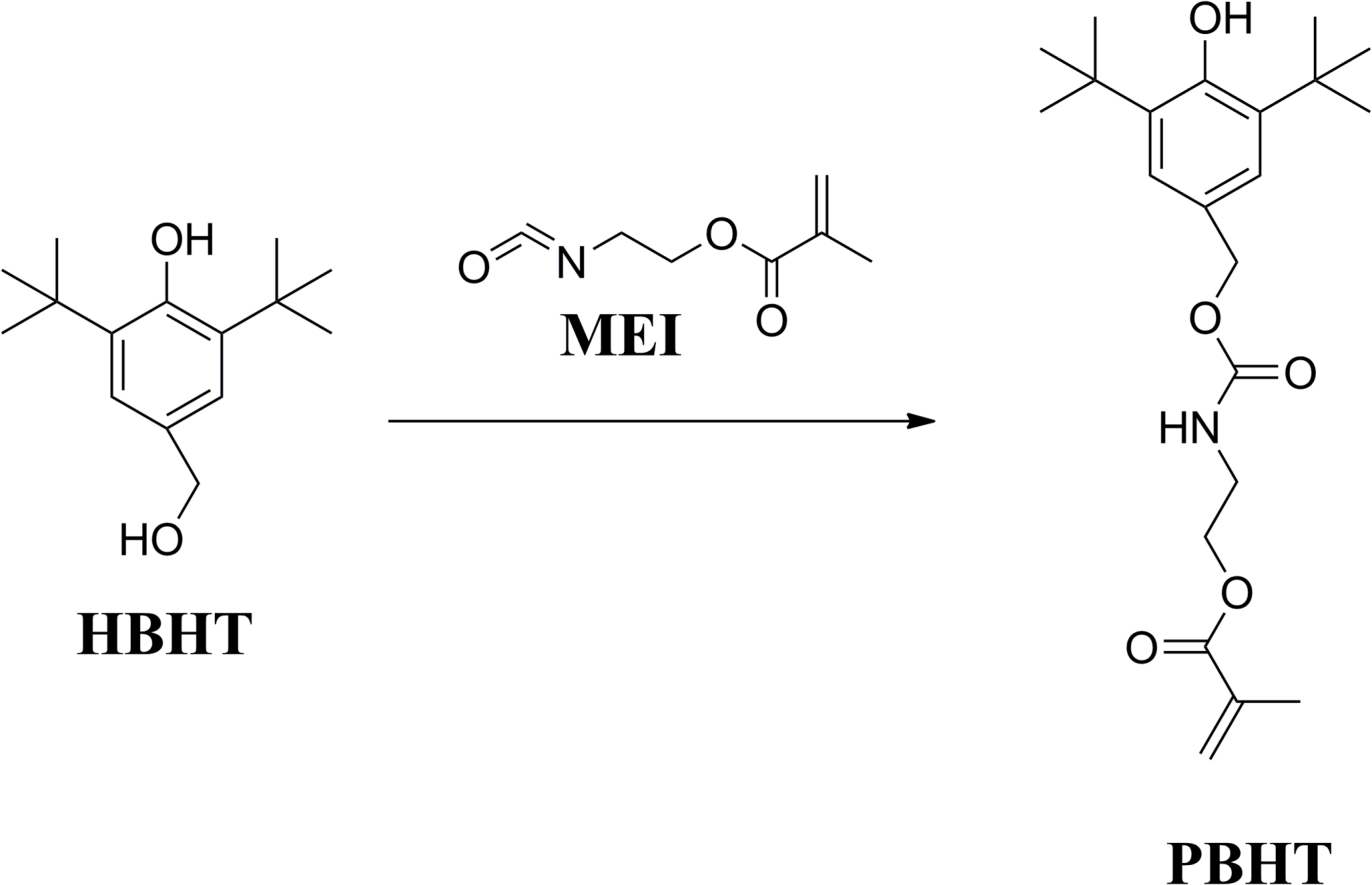
Synthesis route of PBHT

The PBHT was synthesized through the reaction between HBHT and MEI as shown in Fig. 2. Briefly, the mixture of HBHT (23.64 g, 0.10 mol), MEI (15.52 g, 0.10 mol), and a droplet of DBTL in CH_2_Cl_2_ (100 mL) was stirred at 45 °C. The reaction was continued until the infrared absorbance peak of the -NCO group (2270 cm^− 1^) disappeared in the FT-IR (Spectrum One, Perkin-Elmer, Beaconsfield Bucks, UK) spectrum of the sample taken from the reaction medium. After remove the CH_2_Cl_2_, the crude product was washed by diethyl ether. The precipitates were filtered and dried under vacuum at 30 °C overnight to obtain PBHT as yellow powders at a yield of 90%. The structure of PBHT was investigated by FT-IR and ^1^H-NMR (DRX 500, Burker Co., Billercia, MA, USA) spectra. The results of spectroscopic studied for PBHT were as follows: IR (neat): *v* (cm^− 1^) 3642, 2956, 2912, 2868, 1719, 1637, 1436, 1163. 1H-NMR (CDCl_3_-d, *δ*): 7.11 (s, 2 H, Ph-*H*), 6.03 (s, 1H, *trans*, C*H*_2_=C(CH_3_)CO-), 5.51 (s, 1H, cis, C*H*_2_=C(CH_3_)CO-), 5.22 (s, 1H, *H*O-Ph), 5.21 (s, 1H, -CO-N*H*-), 4.94 (s, 2 H, Ph-C*H*_2_-O-), 4.15–4.17 (t, J = 5.0, 5.0 Hz, 2 H, CH_2_-C*H*_2_-O-CO-), 3.44–3.46 (q, J = 4.99, 4.99, 4.94, 2 H, C*H*_2_-CH_2_-O-CO-), 1.86 (s, 3 H, CH_2_=C(C*H*_3_)CO-), 1.37 (s. 18 H, (C*H*_3_)_3_-Ph-(C*H*_3_)_3_).

### Preparation of dental resin composites (DRCs)

Resin matrices of experimental DRCs were prepared according to the proportions shown in Table 1. All components were weighed and mixed well using a magnetic stirrer. Experimental DRCs were prepared by mixing each resin matrix with dental glass fillers in a high-speed mixer (DAC150 FVZ-K, Hauschild, Hamm, Germany) at a speed of 1400 rpm. To minimize heat accumulation, the mixing process was performed in three cycles of 2 min each (total mixing time: 6 min). The temperature during mixing was monitored using an infrared thermometer to ensure that excessive heat buildup did not occur, thereby preventing potential inactivation of the initiation and inhibition systems. The mass ratio between resin matrix and fillers was 1/3 (wt./wt.).

**Table 1:** Components of dental resin matrices for DRCs

Group	Components (wt%)						
	UDMA	Bis-EMA(04)	TEGDMA	BHT	PBHT	CQ	EDB
Control	69.3	19.8	9.9	-	-	0.5	0.5
1% BHT·	68.607	19.602	9.801	1	-	0.495	0.495
2% BHT	67.914	19.404	9.702	2	-	0.49	0.49
3% BHT	67.221	19.206	9.603	3	-	0.485	0.485
1% PBHT	68.607	19.602	9.801	-	1	0.495	0.495
2% PBHT	67.914	19.404	9.702	-	2	0.49	0.49
3% PBHT	67.221	19.206	9.603	-	3	0.485	0.485

### Measurement of double bond conversion

The double bond conversion (DC) of DRCs during and after irradiation were measured using FT-IR with an attenuated total reflectance (ATR) accessory (Spectrum One, Perkin-Elmer, Beaconsfield Bucks, UK). The FT-IR spectra were recorded with 4 scans at a resolution of 4 cm^− 1^. DRCs were analyzed in a 2 mm thick mold with an internal diameter of 4.5 mm. First, the unpolymerized sample was put in the mold and scanned to obtain spectrum before irradiation. Subsequently, the sample was irradiated through an upper glass slide with a visible light-curing unit (Elipar DeepCure-L, 3 M, St. Paul, MN, USA) producing an average irradiance of 1600 mW/cm^2^ (Marc Resin Calibrator, BlueLight Analytics Inc., Canada). The wavelength of the light was between 430 and 480 nm. The sample was irradiated for 40 s and scanned for its FT-IR spectrum every 5 s after the beginning of irradiation. After irradiation, the sample was kept on the ATR accessory for 15 min and scanned to obtain its FT-IR spectrum again. The DC was calculated from the aliphatic C = C peak at 1636 cm^− 1^ and normalized against the aromatic ring C = C peak at 1608 cm^− 1^ according to the formula (1).

1 $$DC\left(t\right)=\frac{{\left(A_{C=C}/A_{ph}\right)}_0-{\left(A_{C=C}/A_{ph}\right)}_t}{{\left(A_{C=C}/A_{ph}\right)}_0}\times100\%$$

where A_c=c_ and A_ph_ are the maximum absorbance peak height of methacrylate C = C at 1636 cm^− 1^ and aromatic ring at 1608 cm^− 1^, respectively. (A_C=C_/A_ph_)_0_ and (A_C=C_/A_ph_)_t_ are the normalized absorbency of the functional group at the irradiation time of 0 and t, respectively. DC (t) is the conversion of methacrylate C = C at a given irradiation time. For each DRC, five trials (*n* = 5) were performed.

### Measurement of shrinkage properties

#### Volumetric shrinkage (VS)

The volumetric shrinkage for each DRC was determined through the variation in density before and after light-curing. The density of sample was measured according to Archimedes’ principle with a commercial density determination kit of the analytical balance (XS105, Mettler Toledo, Greifensee, Switzerland). The mass of the sample was weighed in air and water, and density was calculated according to the Eq. (2)

2 $$\:\mathrm{D=}\frac{{\mathrm{M}}_{\mathrm{a}}\times{\mathrm{D}}_{\mathrm{w}}}{{\mathrm{M}}_{\mathrm{a}}{-\mathrm{M}}_{\mathrm{w}}}$$

where D is the density of the sample, M_a_ is the mass of the sample in air, M_w_ is the mass of the sample in water, and D_w_ is the density of water at the measured temperature. Density of polymerized sample was measured 5 min later after being photo-cured for 40 s. For each DRC, three trials (*n* = 3) were performed respectively to calculate the densities of polymerized and unpolymerized samples. The VS was expressed in % and calculated from the densities according to the Eq. (3)

3 $$\:\mathrm{VS}\mathrm{=}\frac{{\mathrm{D}}_{\mathrm{c}}-{\mathrm{D}}_{\mathrm{u}}}{{\mathrm{D}}_{\mathrm{c}}}\times\:100\%$$

where D_c_ and D_u_ are the densities of polymerized and unpolymerized samples, respectively.

#### Shrinkage stress (SS)

Glass fiber-reinforced composite (FRC) rods with 4 mm diameter and 4 cm in length, had one of their flat surfaces ground with 180 grit silicon carbide sandpaper. Two FRC rods were attached tightly to a universal testing machine (model LRX, Lloyd Instruments Ltd., Fareham, England) and uncured DRC was applied between the FRC rod surfaces. The height of the sample was then set at 1.5 mm. Two light units (Elipar DeepCure-L, 3 M, St. Paul, MN, USA) were used simultaneously for 20 s with the tips in close contact with the DRC sample from both sides to keep the same radiation intensity as double bond conversion measurement. Contraction forces were monitored for 3 min at room temperature (22 °C). The SS was calculated by dividing the shrinkage force by the cross-section area of the FRC rod. The maximum SS value was taken from the plateau at the end of the SS/time curve. Six samples (*n* = 6) were tested for each DRC.

### Mechanical tests

#### Measurement of flexural strength (FS) and modulus (FM)

Three-point bending test samples (2 × 2 × 25 mm) were made from each DRC. Bar-shaped samples were made in half-split stainless steel molds between transparent Mylar sheets. The DRC was light-cured using a hand light-curing unit (Elipar DeepCure-L, 3 M, St. Paul, MN, USA). Each of the five overlapping portions was individually light-cured for 20 s from one side of the metal mold. Eight samples (*n* = 8) were prepared for each DRC. All the samples were stored dry at 37 °C for one day before testing. The three-point bending test was carried out on a universal material testing machine (Model LRX; Lloyd Instruments Ltd, AMETEK, West Sussex, UK) according to ISO 4049 (test span: 20 mm, cross-head speed: 1 mm/min, indenter: 2 mm diameter). The values of FS and FM were obtained directly from the software of the machine (Nexygen 4.0, Lloyd Instruments Ltd., Fareham, England) according to Eqs. (4) and (5).

4 $$\:\mathrm{F}\mathrm{S}=\frac{3\mathrm{P}\mathrm{L}}{2\mathrm{b}{\mathrm{h}}^{2}}$$

5 $$\:\mathrm{F}\mathrm{M}=\frac{\mathrm{S}{\mathrm{L}}^{3}}{4\mathrm{b}{\mathrm{h}}^{3}}$$

where P is the maximum load (N), L is the span length (20 mm), b is the specimen width, and h is the thickness of the specimen (mm). S is the stiffness (N·m^− 1^). S = F/d, where d is the deflection corresponding to load F at a point in the straight-line portion of the trace.

#### Measurement of fracture toughness (FT)

Single-edge-notched-beam samples (2.5 × 5 × 25 mm) were prepared to determine fracture toughness according to an adapted ISO 20795-2 standard method (ASTM 2005). A custom-made stainless steel split mold was used, which enabled the specimen’s removal without force. An accurately designed slot was fabricated centrally in the mold extending until its mid-height, which enabled the central location of the notch and optimization of the crack length (x) to be half of the specimen’s height. The DRC was inserted into the mold placed over a Mylar-strip-covered glass slide in one increment. A sharp and centrally located crack was produced by inserting a straight edged steel blade into the prefabricated slot before polymerization. The DRC was light cured for 20 s in five separate overlapping portions. The upper side of the mold was covered with a Mylar strip and a glass slide from both sides of the blade, before being exposed to the light. Upon the removal from the mold, each sample was polymerized also on the opposite side. Six samples (*n* = 6) were prepared for each DRC. The samples were stored were stored dry at 37 °C for one day before testing. The samples were tested in three-point bending mode, in a universal material testing machine at a crosshead speed of 1.0 mm/min.

The FT was calculated using the Eq. (4)

6 $$\:{\mathrm{K}}_{\mathrm{max}}\mathrm{=}\frac{\mathrm{PL}}{\mathrm{B}}{\mathrm{W}}^{\mathrm{3/2}}f\left(x\right)$$

where *f* is a geometrical function dependent on x:

7 $$\begin{array}{c}f\left(x\right)=3x^{1/2}\lbrack1.99-x\left(1-x\right)\\(2.15-3.93x+2.7x^2\rbrack/\lbrack2\left(1+2x\right){(1-x)}^{3/2}\rbrack\end{array}$$

Here P is the maximum load in kilonewtons (kN), L is the span length (20 mm), B is the sample thickness in centimeter (cm), W is the sample width (depth) in cm, x is a geometrical function dependent on a/W and a is the crack length in cm.

### Measurement of water sorption (WS) and solubility (SL)

According to ISO 4049:2019(E), the DRC was filled into a 1 mm thick cylindrical steel mold with an internal diameter of 15 mm between transparent Mylar sheets, and then light-cured using a hand light-curing unit (Elipar DeepCure-L, 3 M, St. Paul, MN, USA) for 20 s on nine separated overlapping portions. Five samples (*n* = 5) were prepared for each DRC. The initial dry weight of every sample was weighed by an analytical balance (XS105, Mettler Toledo, Greifensee, Switzerland) with an accuracy of 0.01 mg and denoted as M_i_, then every sample was immersed in 30 mL of distilled water and stored at 37 °C for 7 days. After that, samples were taken out, blotted dry to remove excess water and weighed to get mass after water immersion M_a_. Finally, the samples were dried at 60 °C in a desiccator and weighed until getting a constant mass (M_f_). The WS and SL were then calculated according to Eqs. (6) and (7)

8 $$\:WS=\frac{{M}_{a}-{M}_{f}}{V}$$

9 $$\:SL=\frac{{M}_{i}-{M}_{f}}{V}$$

where V is the volume of the sample.

### Measurement of leachable inhibitors in DRCs

The total amount of leachable inhibitors in DRCs was investigated using Gas chromatography-mass spectrometry (GC-MS) (Agilent 8890-7000D, Agilent Technologies, Santa Clara, CA, USA) with selected ion monitoring (SIM) method. The SIM parameters for each analyte were as follows: for BHT, retention time = 13.34 min, quantifier ion m/z = 205, qualifier ion m/z = 220; for PBHT, retention time = 15.26 min, quantifier ion m/z = 235, qualifier ion m/z = 250.

0.7439 g of N-pentanol was added in a 20 mL volumetric flask, and then methanol was added in the flask to 20 mL, getting an internal standard solution. For making calibration curve, BHT or PBHT was dissolved in methanol with a series of concentration. 100 µL of internal standard solution was added in a 10 mL volumetric flask, and then BHT or PBHT solution was added in the flask to 10 mL. 3 mL of solution was taken out of the flask and analyzed in the GC-MS.

The limits of detection (LOD) and quantification (LOQ) were determined by the signal-to-noise ratio method using spiked blank matrix samples. Based on injected standards (BHT: 79.75 µg/mL, S/*N* = 155; PBHT: 41.75 µg/mL, S/*N* = 54), LOD/LOQ were calculated as 1.54/5.15 µg/mL for BHT and 2.32/7.72 µg/mL for PBHT.

1 g of DRC was light cured for 40 s and stored in darkness at 37 °C for 24 h. Afte that, the cured DRC was immersed in 20 mL of methanol and stirred magnetically for 3 days. 15 mL of solution was then taken out and centrifuged to remove precipitates. 100 µL of internal standard solution was added in a 10 mL volumetric flask, and then the centrifuged solution was added in the flask to 10 mL. 3 mL of solution was taken out of the flask and analyzed in the GC-MS. Every DRC was tested three times (*n* = 3).

### Statistical analysis

Two-way ANOVA analysis was used to examine statistical differences between variables by employing GraphPad Prism 8 (GraphPad Software, Inc., USA). Prior to ANOVA, the assumptions of normality and homogeneity of variances were verified using Shapiro–Wilk tests and Levene’s tests, respectively, and both assumptions were met (*p* > 0.05 for all comparisons). Tukey’s multiple comparisons test (at a significance level of *p* = 0.05) was used to compare properties of DRCs with the same inhibitor and Sidak’s multiple comparisons test was used to compare properties of DRCs with different inhibitors at the same concentration (at a significance level of *p* = 0.05).

## Results

**Fig. 3:**
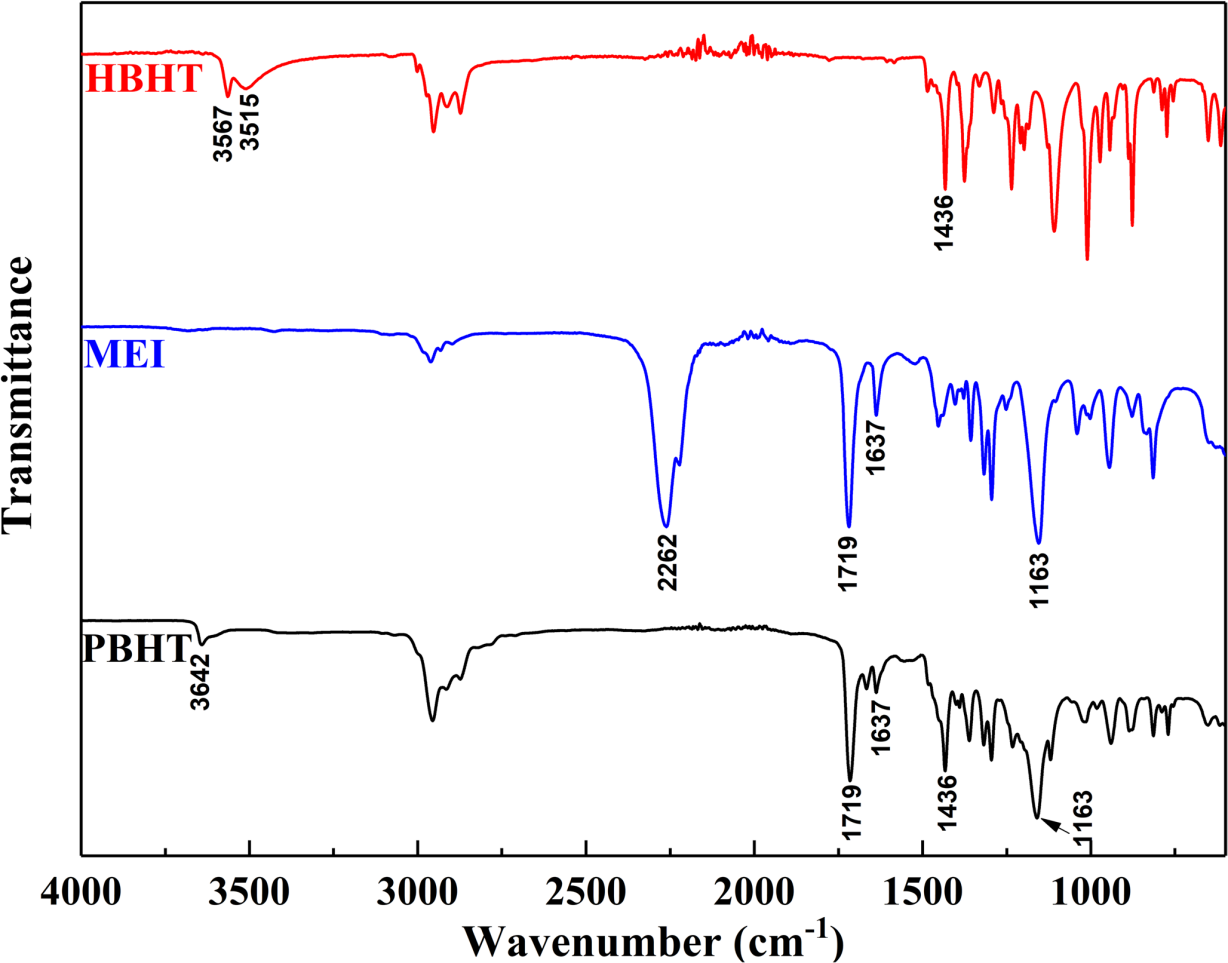
FT-IR spectra of HBHT, MEI, and PBHT

**Fig. 4:**
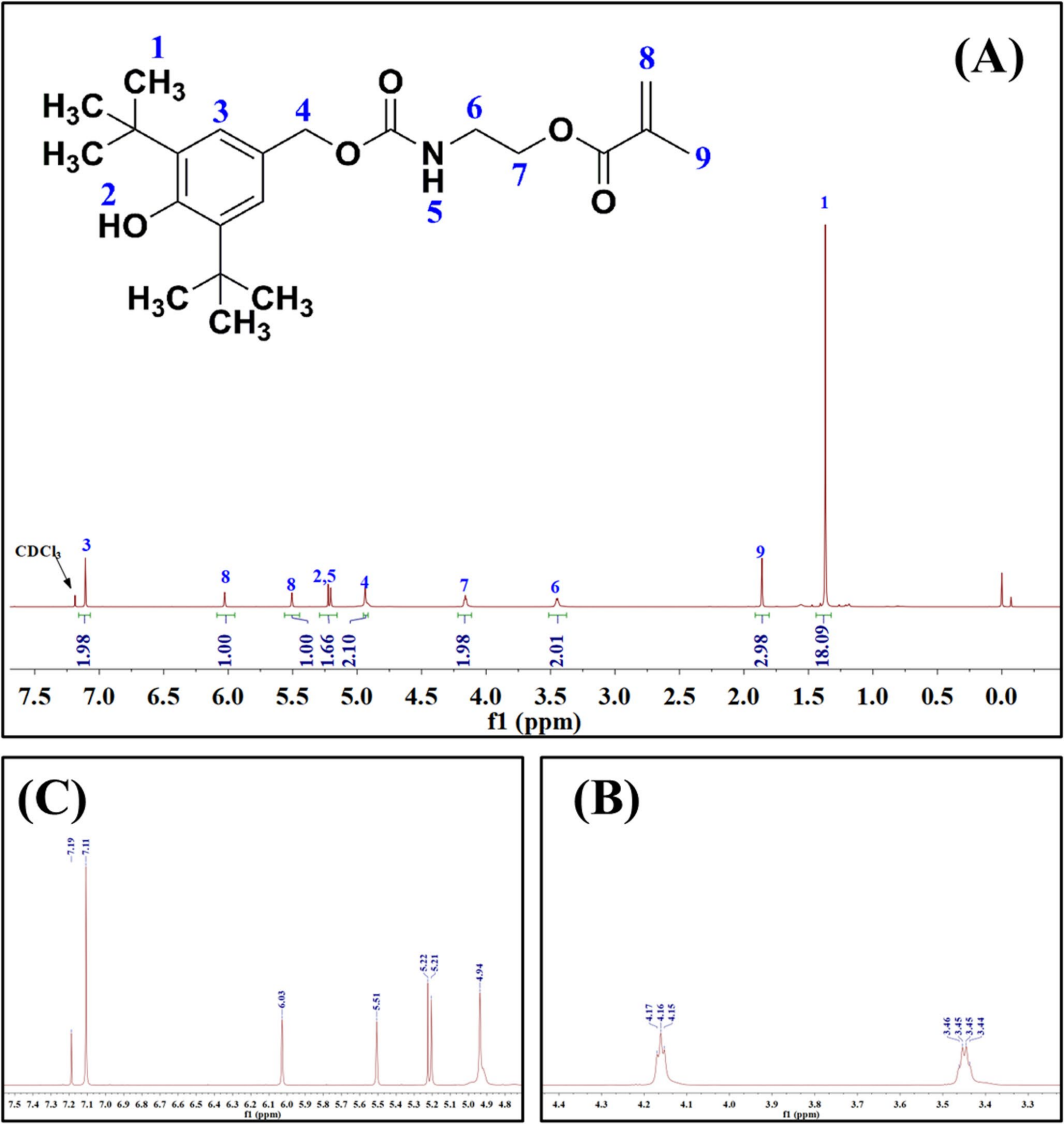
^1^H-NMR spectrum of PBHT: (**A**) The whole spectrum; (**B**) Zoom spectrum between shift at 3.4 to 4.3 ppm; (C) Zoom spectrum between shift at 4.8 to 7.3 ppm

The FT-IR and ^1^H-NMR spectra of synthesized inhibitor PBHT were shown in Figs. 3 and 4, respectively. The disappearance of isocyanate peak at 2262 cm^− 1^ (Fig. 3) together with the appearance of absorbance peaks at 1719 cm^− 1^ and 1637 cm^− 1^ (Fig. 3) in the PBHT indicated that the reaction between HBHT and MEI was successfully carried out. The chemical shifts of protons and the relative area of each peak (Fig. 4) revealed that the structure of PBHT was exactly the same as designed.

**Fig. 5:**
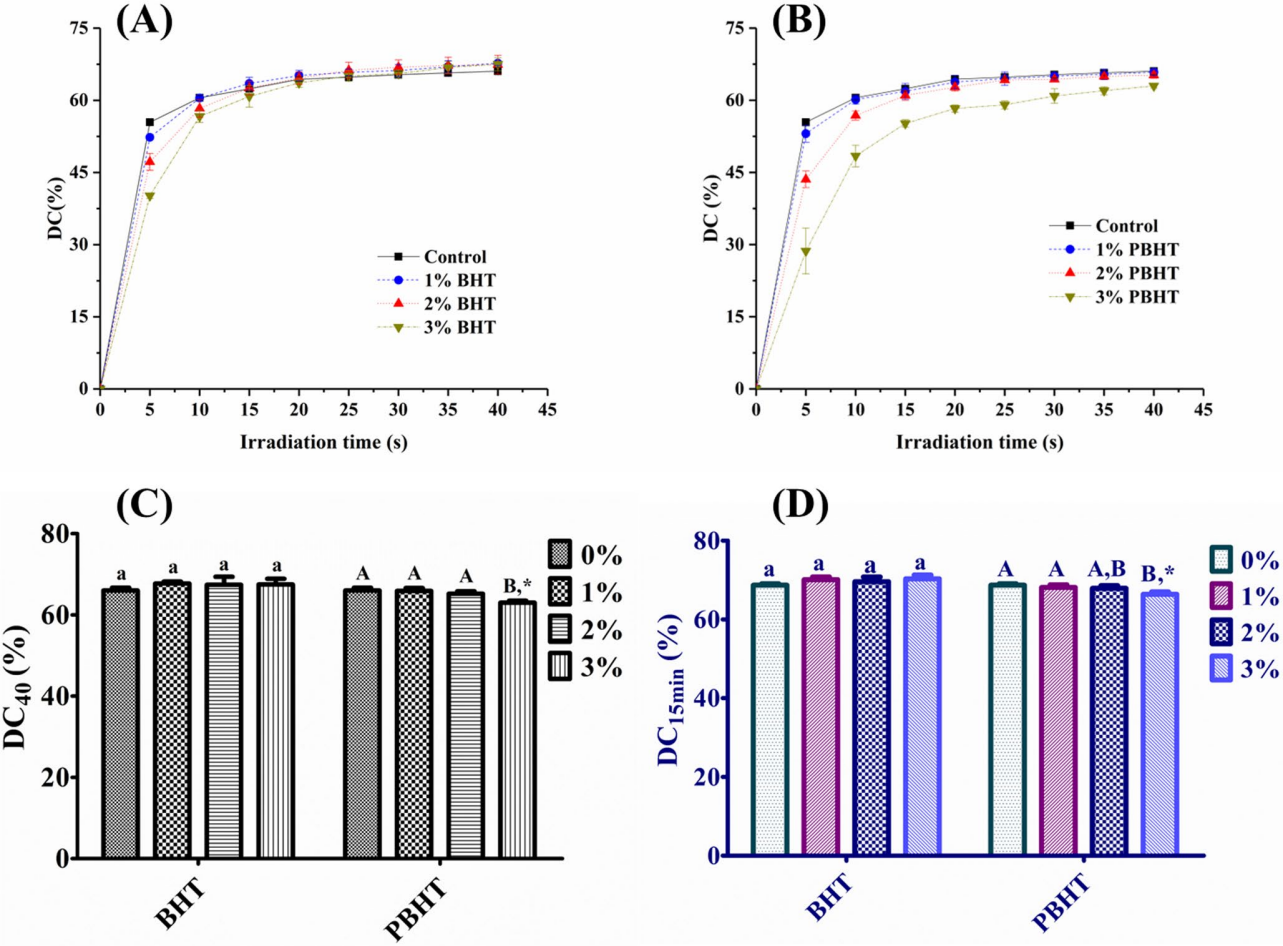
The curves of double bond conversion versus irradiation time of dental resin composites with BHT (**A**) or PBHT (**B**), and the final double bond conversion at different time: (**C**) after 40 s of irradiation; (**D**) 15 min after stopping irradiation. ^a^ The same lowercase letter indicates that there is no significant difference in double bond conversion between groups with different fractions of BHT in resin matrix (*p* > 0.05). ^A^ The same uppercase letter indicates that there is no significant difference in double bond conversion between groups with different fractions of PBHT in resin matrix (*p* > 0.05). ^*^ The asterisk indicates the statistical differences in double bond conversion between groups with different inhibitors at the same fraction in resin matrix (*p* < 0.05)

The results of double bond conversion (DC) and curves of DC versus irradiation time were shown in Fig. 5. The polymerization was delayed (Fig. 5(A) and (B)) upon the addition of inhibitors to the DRCs. At concentrations of 2 wt% and 3 wt% in the resin matrix, the PBHT showed a more pronounced delaying effect compared to the BHT. All the DRCs had DC higher than 60%, and only DRC with 3 wt% of PBHT in resin matrix had significant lower DC (*p* < 0.05) than the control (Fig. 5(C) and (D)).

**Fig. 6:**
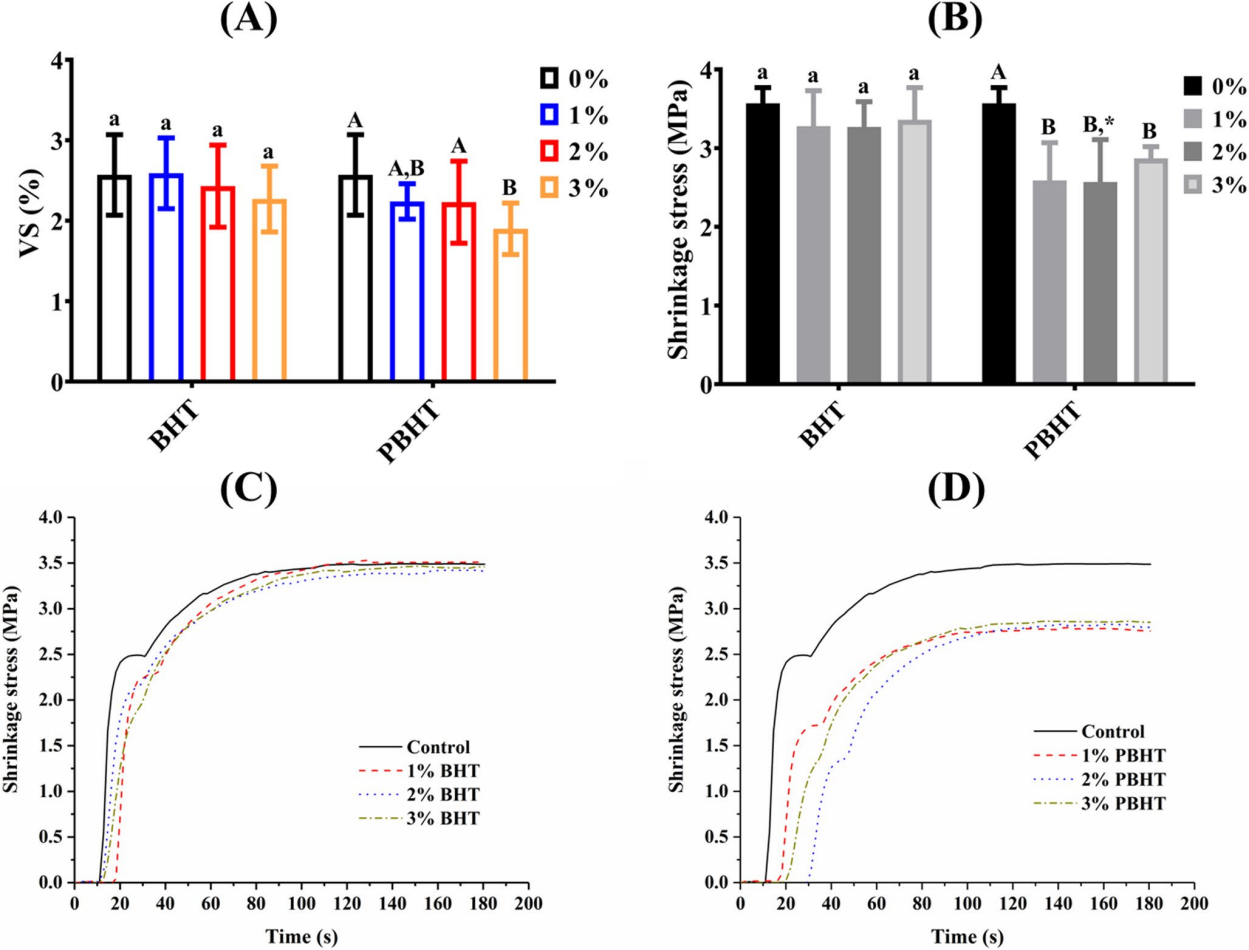
The values of volumetric shrinkage (**A**) and shrinkage stress (**B**) of experimental dental resin composites, and curves of shrinkage stress versus time of dental resin composites with BHT (**C**) or PBHT (**D**). ^a^ The same lowercase letter indicates that there is no significant difference in volumetric shrinkage/shrinkage stress between groups with different fractions of BHT in resin matrix (*p* > 0.05). ^A^ The same uppercase letter indicates that there is no significant difference in volumetric shrinkage/shrinkage stress between groups with different fractions of PBHT in resin matrix (*p* > 0.05). ^*^ The asterisk indicates the statistical differences in volumetric shrinkage/shrinkage stress between groups with different inhibitors at the same fraction in resin matrix (*p* < 0.05)

The data in Fig. 6 showed that the addition of BHT to the resin matrix of DRCs did not result any significant variation (*p* > 0.05) in volumetric shrinkage (VS) and shrinkage stress (SS). In the contrast, the addition of PBHT significantly reduced SS (*p* < 0.05), and only DRC with 3 wt% of PBHT in the resin matrix had significant lower VS (*p* < 0.05) than the control. The maximum shrinkage stress rates of experimental DRCs were listed in Table 2. The development of shrinkage stress was significantly slowed by adding inhibitors (*p* < 0.05), the maximum shrinkage stress rate was in a trend of decreasing with the increasing of inhibitor concentration.

**Table 2:** Maximum shrinkage stress rate, flexural properties, water sorption and solubility of experimental DRCs

DRCs	Properties					
	Maximum shrinkage stress rate (×10^− 2^MPa·s^− 1^)	FS (MPa)	FM (GPa)	FT (MPa·m^1/2^)	WS (µg/mm^3^)	SL (µg/mm^3^)
Control	47.5 ± 2.8^a, A^	133 ± 20^a, A^	12.08 ± 1.12^a, A,B^	1.13 ± 0.08^a, A^	15.91 ± 0.61^a, A,B^	2.92 ± 0.13^a, A^
1%BHT	34.8 ± 5.1^b^	128 ± 15^a^	13.12 ± 1.55^a, b^	1.14 ± 0.09^a^	16.10 ± 0.35^a^	4.83 ± 0.14^b^
2%BHT	27.9 ± 2.4^b^	144 ± 19^a^	12.48 ± 1.02^a, b^	1.11 ± 0.10^a^	16.24 ± 0.53^a^	5.36 ± 0.29^b^
3%BHT	21.8 ± 2.6^c^	111 ± 21^a^	14.16 ± 1.36^b^	1.16 ± 0.07^a^	16.81 ± 0.38^a^	6.71 ± 0.35^c^
1%PBHT	30.8 ± 3.9^B^	136 ± 19^A^	13.11 ± 1.24^A^	1.16 ± 0.11^A^	15.30 ± 0.25^A, *^	4.98 ± 0.18^B^
2%PBHT	19.8 ± 2.0^C, *^	125 ± 22^A^	11.55 ± 1.57^A, B^	1.19 ± 0.09^A^	16.36 ± 0.41^A, B^	4.57 ± 0.29^B, *^
3%PBHT	13.1 ± 1.5^D, *^	134 ± 14^A^	10.34 ± 1.69^B, *^	1.12 ± 0.10^A^	16.99 ± 0.57^B^	5.89 ± 0.17^C, *^

^a^ The same lowercase letter indicates that there is no significant difference in properties between groups with different fractions of BHT in resin matrix (*p* > 0.05)

^A^ The same uppercase letter indicates that there is no significant difference in properties between groups with different fractions of PBHT in resin matrix (*p* > 0.05)

^*^ The asterisk indicates the statistical differences between groups with different inhibitor at the same fraction in resin matrix (*p* < 0.05)

The flexural strength (FS), modulus (FM), fracture toughness (FT), water sorption (WS), and solubility (SL) of experimental DRCs were summarized in Table 2. Compared with the control group, all inhibitor-containing DRCs had comparable FS and FM (*p* > 0.05), except for 3%BHT, which had higher FM (*p* < 0.05) than the control. All DRCs had similar FT (*p* > 0.05). The mean values of WS and SL showed an increasing trend with the increase of inhibitor’s fraction in the resin matrix. Compared with the control group, all of inhibitors containing DRCs showed similar WS (*p* > 0.05). When the inhibitor fraction exceeded 1 wt%, the SL of PBHT-containing DRCs was lower (*p* < 0.05) than that of BHT-containing DRCs.

**Fig. 7:**
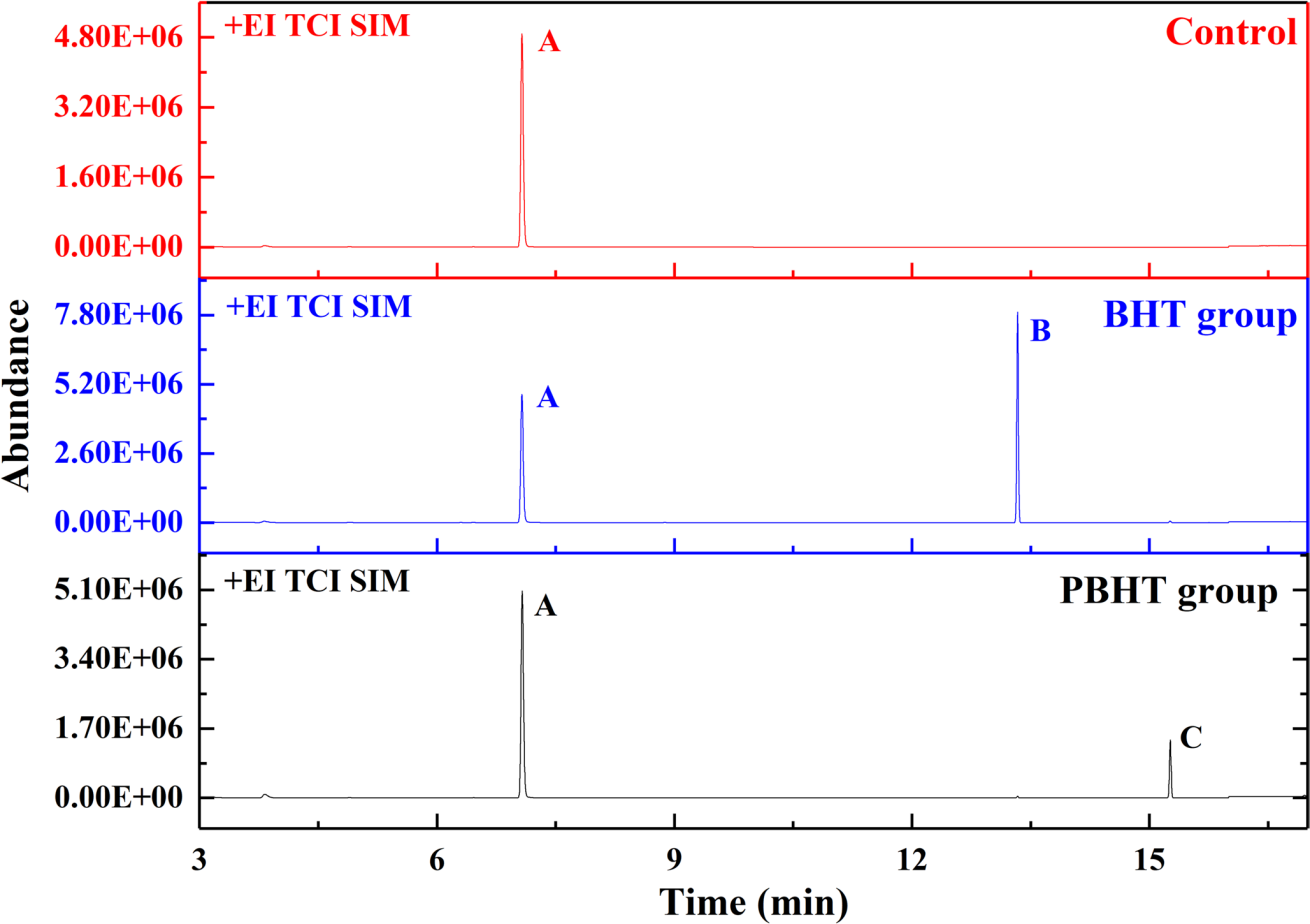
Gas chromatography spectra with selected ion of experimental dental resin composites

The GC-MS spectra of DRCs with and without inhibitors were shown in Fig. 7, and the amounts of leachable inhibitors in DRCs were listed in Table 3. The results showed that the amount of leachable inhibitors increased (*p* < 0.05) with the increasing of inhibitors content in the resin matrix of DRCs. Meanwhile, with the same inhibitors content in the resin matrix, the DRCs with PBHT exhibited less amount of leachable inhibitors (*p* < 0.05) than the DRCs with BHT.

**Table 3:** Amount of leachable inhibitors in every DRCs

DRCs	Amount of leachable inhibitors (µg/g)
Control	-
1%BHT	549 ± 3^a, *^
2%BHT	1102 ± 29^b, *^
3%BHT	2105 ± 7^c, *^
1%PBHT	132 ± 7^A^
2%PBHT	290 ± 15^B^
3%PBHT	717 ± 22^C^

^a^ The same lowercase letter indicates that there is no significant difference in amount of leachable inhibitors between groups with different fractions of BHT in resin matrix (*p* > 0.05)

^A^ The same uppercase letter indicates that there is no significant difference in amount of leachable inhibitors between groups with different fractions of PBHT in resin matrix (*p* > 0.05)

^*^ The asterisk indicates the statistical differences in amount of leachable inhibitors between groups with different inhibitor at the same fraction in resin matrix (*p* < 0.05)

## Discussion

In previous studies, the addition of BHT to DRCs was shown to reduce shrinkage stress effectively. This reduction was achieved as BHT quenched free radicals, slowing the polymerization rate and allowing molecular rearrangement, thus lowering the shrinkage stress [24, 30, 39]. The influence of BHT on the final degree of conversion (DC) of DRCs varied across different studies. Braga et al. [24] found that the concentration of BHT (ranging from 0.05 to 1.0 wt%) had no influence on DC, whereas Nassar et al. [39] and Pereira et al. [30] reported that the final DC could be reduced when the BHT concentration reached 0.5 wt% or higher. These discrepancies may be related to the differences in matrix composition and the amount of fillers [39]. In this study, the addition of BHT was found to slow polymerization (Fig. 5(A)) and delay the development of shrinkage stress (Fig. 6(C)). However, no effect on the final DC (Fig. 5(C) and (D)) or shrinkage stress (Fig. 6(B)) of DRCs was observed, even at BHT concentrations of up to 3 wt% in the resin matrix. This should be attributed to the high concentration of UDMA in the resin matrix, which was much significantly greater than that in other studies [24, 30]. The abundant hydrogen bonds formed by the -NH- groups in UDMA could enhance the propagation efficiency by pre-organizing the double bonds and increase the reactivity of the propagating radicals through resonance effects [40], leading to a rapidly achieved gelation stage. Consequently, most of the BHT may become trapped within the dense network formed at an early stage, thereby showing a reduced inhibitory effect. Moreover, the intrinsic reactive of the UDMA/Bis-EMA/TEGDMA network, higher light intensity and longer extended irradiation time compared with other studies [24, 30] also might be the reason for the similar DC.

Unlike BHT, PBHT contains one methacrylate group in its structure, enabling it to polymerize with other monomers in the matrix. The PBHT could delay gelation stage not only by consuming radicals but also by reducing the cross-link density, owing to its mono-methacrylate structure [40]. As a result, the inhibitory effect of PBHT could persist for an extended period, quenching more radicals. Therefore, at the same concentration (2 wt% and 3 wt%) in the resin matrix, PBHT was much more effective at slowing the polymerization rate than BHT (Fig. 5(A) and (B)). This led to a lower DC in DRCs with 3 wt% PBHT in the resin matrix (Fig. 5(C) and (D)) and reduced shrinkage stress in PBHT-containing DRCs (Fig. 6(B) and (D)). Together with the lower polymerization rate, the mono-functional PBHT could reduce the cross-link density of DRCs, which might be also benefit for dissipating more stress during polymerization. On the other hand, if PBHT polymerizes before quenching free radicals, its mobility could be reduced relative to BHT. However, according to the findings of this study, this effect is negligible. Future research should therefore compare the reaction kinetics of the double bond in PBHT with radicals and that of the phenolic hydrogens in PBHT with radicals. Compared with the control group, only DRC with 3 wt% PBHT in resin matrix exhibited lower volumetric shrinkage, which was mainly attributed to its lower DC [41]. Given the low curing rates observed in PBHT-containing DRCs, the extent of oxygen inhibition may be altered. However, this potential interaction was not directly investigated in the present study and warrants further investigation. It should be noted that volumetric shrinkage was measured at 5 min after the start of irradiation, representing early-stage polymerization shrinkage. Post-cure polymerization may continue beyond this period, so the reported values reflect early rather than final shrinkage. Nevertheless, all groups were measured under identical conditions, allowing valid comparison of material effects.

High concentrations of BHT were reported to reduce flexural strength (FS) and modulus (FM) of DRCs [30, 39], resulting from the low cross-link density induced by reduced DC [42]. In this research, the addition of BHT (from 1 wt% to 3 wt%) and PBHT (1 wt% and 2 wt%) in the resin matrix had no significant influence on DC, no deterioration in FS and FM was observed in these inhibitor-containing DRCs. For DCR with 3 wt% of BHT in the resin matrix, a visible reduction in FS was observed, although the difference did not reach statistical significance (*p* > 0.05). This should be due to the overload of BHT. Although the DC of DRC with 3 wt% PBHT in the resin matrix was slightly lower (66.4 ± 0.6%) than the control (68.7 ± 0.7%), the absolute difference was only 2.3%. Given this small magnitude, this minor reduction in DC is unlikely to have significantly altered the mechanical properties. Accordingly, no significant differences in FS and FM were observed between these two groups. As the FT values of DRCs have been reported to be dependent mainly on the reinforcing filler system and the adhesion of between filler particles and the resin matrix [43, 44], it follows that all experimental DRCs in this study exhibited comparable FT values.

Although the addition of BHT was reported to have no significant influence on the water sorption (WS) of DRCs [30], the maximum concentration used in that study was only 1 wt%. In our work, the addition of 1 wt% or 2 wt% inhibitors to the resin matrix also showed no effect on WS compared to the control. However, when the inhibitor concentration was increased to 3 wt%, an increase in WS was observed. This indicates that an excessive amount of inhibitors can raise the WS of DRCs, which is undesirable because water intrusion may lead to deterioration of mechanical properties and reduce the service life of DRCs [45, 46].

It was reported that within the first 7 days, the main substance eluted from DRCs was unreacted monomers [46, 47], and the amount of which was negatively correlated with the DC [48]. In this study, except for the DRC with 3 wt% of PBHT in the resin matrix, which showed a lower DC than control, all the other inhibitor-containing DRCs exhibited comparable DC as the control. However, the SL of all inhibitor-containing DRCs were significantly higher than that of the control, and SL increased with the increasing of inhibitor concentration. The solubility of DRCs is governed not only by the DC, but also by the leachable additives and cross-link density of the polymeric network. As an unpolymerizable small compound, BHT can be easily eluted from the DRC, thus BHT-containing DRCs had higher SL than the control. Although PBHT can participate in polymerization, its mono-methacrylate structure would lead to more residual unreacted monomers and a lower cross-linking density at the same DC. Consequently, PBHT-containing DRCs also showed higher SL than the control. However, because PBHT is polymerizable, at the same concentration in the resin matrix, the PBHT-containing DRCs demonstrated lower SL than the BHT-containing DRCs. Even though, WS and SL of all experimental DRCs still met the requirements in ISO standard, which should be less than 40 µg/mm^3^ and 7.5 µg/mm^3^, respectively. Clinically, the increased solubility may compromise dimensional stability and biocompatibility of DRCs. Therefore, further work is required to assess long-term safety and durability of these materials. Based on the above results, the first hypothesis of this study can be partially accepted.

As hypothesized, abundant leachable BHT existed in the BHT-containing DRCs. In contrast to BHT, PBHT can copolymerize with other monomers, thus less leachable PBHT was detected in the PBHT-containing DRCs. Therefore, the second hypothesis of this study can be accepted. However, the lower crosslinking density induced by PBHT could increase the leachable fraction of unreacted monomers like UDMA, Bis-EMA, and TEGDMA, as the PBHT containing DRCs exhibited higher water solubility than the control. Direct quantification of monomers elution should be performed in future studies.

This study is subject to several limitations. Key properties such as cytotoxicity, mechanical performance under water storage, wear resistance, and in vitro tooth performance of PBHT-containing DRCs were not investigated and should be addressed in future studies. In addition, the higher concentration groups were primarily intended for experimental comparison and mechanistic evaluation rather than direct clinical application, and therefore their translational relevance should be interpreted with caution.

## Conclusion

With the limitation of this research, it could be concluded that the synthesized polymerizable inhibitor PBHT (2,6-di-tert-butyl-4-(methacryloyloxy-ethyl-carbamate-methyl)phenol) could reduce shrinkage stress of dental resin composites without influencing physicochemical properties except water sorption and solubility. Compared with commercial inhibitor butylated hydroxytoluene, PBHT could polymerize with monomers in resin matrix, leaving less leachable inhibitors in dental resin composites. The optimal concentration of PBHT in the resin matrix was 1 wt%, and the dental resin composite containing this concentration exhibited the best comprehensive properties among all formulations tested.

## Data Availability

No datasets were generated or analysed during the current study.
